# An exploratory study of CT-guided percutaneous radiofrequency ablation for stage I thymoma

**DOI:** 10.1186/s40644-019-0267-8

**Published:** 2019-12-02

**Authors:** Jun Dong, Shaofei Yuan, Boyang Chang, Jinsheng Huang, Xiaojing Geng, Xiuyu Cai, Pili Hu, Bei Zhang, Liangping Xia, Peihong Wu

**Affiliations:** 1 Department of Integrated Therapy in Oncology, State Key Laboratory of Oncology in South China, Collaborative Innovation Center for Cancer Medicine, Sun Yat-sen University Cancer Center, Guangzhou, 510060 People’s Republic of China; 2 Department of Oncology, The Third Affiliated Hospital of Wenzhou Medical University, Wansong Road, Ruian, 108 China; 3 Department of Vascular Interventional Radiology, the Third Affiliated Hospital of Sun Yat-sen University, Guangzhou, 510630 People’s Republic of China; 4 Department of Geriatric Medicine, the Fifth Affiliated Hospital of Sun Yat-sen University, Guangzhou, 510630 People’s Republic of China; 5 Department of Medical Imaging & Image Guided Therapy, State Key Laboratory of Oncology in South China, Collaborative Innovation Center for Cancer Medicine, Sun Yat-sen University Cancer Center, Guangzhou, 510060 People’s Republic of China

**Keywords:** CT-guided, Percutaneous radiofrequency ablation, Early stage, Thymoma

## Abstract

**Background:**

Thymoma is a rare tumor that originates from thymic epithelial cells and is usually associated with myasthenia gravis. Radiofrequency ablation (RFA) is a minimally invasive and curative treatment for other tumors, but RFA has not been used for the early treatment of thymoma.

**Methods:**

The current study included 13 patients with stage I thymoma who were not candidates for surgical resection or video-assisted thoracoscopic surgery (VATS). All patients underwent first-line CT-guided percutaneous RFA. The feasibility and therapeutic effects of the intervention were thoroughly documented.

**Results:**

All tumors were completely ablated (13 / 13, 100%). During follow-up (median 80.5 months, range, 64.6–116.9 months), only 1 of the 13 patients had recurrence of thymoma (1 / 13, 7.7%) at 35.5 months after the initial ablation. There were no surgery-related deaths after RFA treatment. The most common complications were fever (13 / 13, 100%) and pain (13 / 13, 100%). There was only one patient who occurred severe puncture-related bleeding during the procedure that needed blood transfusion and intravascular embolization of the punctured-injured vessel.

**Conclusion:**

CT-guided percutaneous RFA for treatment of stage I thymoma is associated with minor trauma, few complications and good treatment outcomes.

## Introduction

Thymoma is most commonly found in the anterior mediastinum and is the third most commonly found tumor in the mediastinum. Thymoma originates from the epithelial cells of the thymus. Typically, thymoma is considered cytologically benign and characterized by slow growth, local invasion and uncommon extrathoracic metastases. According to the statistics, the incidence of thymoma was reported at only 0.17/100,000 per year in Europe and 0.15/100,000 per year in the United States [1, 2]. However, approximately 30 to 50% of thymoma patients suffer from myasthenia gravis (MG), which is an autoimmune disease of the neuromuscular junction mediated primarily by anti-acetylcholine receptor antibodies. MG limits muscle movement. In extremely cases, MG even affects the muscles that control breathing, which might eventually lead to patient death. Surgical resection is the only recommended curable treatment for diagnosed thymoma. As reported, the 5-year overall survival rate of stage I thymoma patients was 90% in patients who underwent complete resection, 97.3% in patients who underwent thymectomy and 96.9% in patients who underwent thymothymectomy [3, 4]. Although thymectomy has been a standard procedure based on several retrospective studies, some researchers have noted that surgical treatment might not be optimal for some patients with thymoma [4–8]. In addition, trauma to the thoracic diaphragm and movement after traditional open-chest surgery could easily lead to postoperative atelectasis, pneumonia, respiratory dysfunction, and other severe complications, causing patients to recover slowly, extend their hospital stays and incur increased costs. Although video-assisted thoracoscopic surgery is increasingly used for mediastinal tumor resection and has the advantages of minor trauma and a clear surgical field, the surgery is complex, difficult, and highly technical to perform [9–12]. Therefore, the application of surgical resection for thymoma treatment is still controversial.

With the continuous development of medical imaging technology in recent years, percutaneous radiofrequency ablation has emerged as a curative minimally invasive therapy for many types of carcinoma, especially hepatic carcinoma [13]. RFA is an effective, minimally invasive modality for carcinoma treatment that is associated with little damage, low costs, and accurate ablation of the masses. However, there is only one case report about RFA being applied in advanced-stage thymomas as an adjuvant therapy [14]. In addition, there is no research on the use of RFA as a treatment for early-stage thymoma. Additionally, the 2016 NCCN guidelines did not include RFA as a treatment [15]. To investigate the feasibility and effectiveness of RFA for thymomas, we performed first-line CT-guided percutaneous radiofrequency ablation in patients diagnosed with stage I thymomas.

## Materials and methods

### Study population

The current research was approved by the Institutional Review Board of our hospital, and all the procedures met the basic standards of the Declaration of Helsinki. All patients were completely informed of the potential risks and benefits of the procedure before undergoing the procedure. All patients stated their understanding of the procedure, had all their questions answered and voluntarily signed the informed consent form.

Between February 1, 2001 and December 20, 2011, a total of 215 patients were diagnosed with thymoma in our hospital. In total, 13 patients received first-line CT-guided RFA. The clinicopathological features of the patients are shown in Table 1.

**Table 1:** Clinicopathological features of the patients with thymoma

Features	Number (*n* = 13)	Percentage (100%)
Median age (range, *years*)	36.1 (12–57)	
Gender (*n*)		
Male	6	46.2
Female	7	53.8
Myasthenia gravis (*n*)		
Yes	6	46.2
No	7	53.8
Median size (range, mm)	24.5 (16.3–33.5)	
WHO Histological type (*n*)		
A	6	46.1
B1	3	23.1
AB	4	30.8

### Diagnosis

The patients were initially diagnosed with stage I thymomas using enhanced-contrast computed tomography (CT) or magnetic resonance imaging (MRI) according to standard CT and MRI criteria. Fine-needle cytology (FNC) was essential for the diagnosis before the treatment. The size and infiltration of the thymomas were calculated according to the CT or MRI images.

### Enrollment criteria

Patients who were potential candidates for first-line CT-guided RFA were as follows: (1) patients with stage I thymomas according to the Masaoka staging system [16]; (2) patients with an East Coast Oncology Group (ECOG) performance status value ≤2; (3) patients who were not considered high-risk for anesthesia and surgery; (4) patients with normal coagulation function without bleeding tendency; and (5) patients who strongly refused any other further treatment but voluntarily accepted RFA after being informed of the potential risks and benefits.

### Patient preparation

A routine blood count, serum liver function test, renal function test and assessment of prothrombin time were performed prior to RFA. To reduce the risk of complications associated with intravenous anesthetics, NPO 12 h (nothing by mouth) was required before the procedure.

### Puncture paths design

**Fig. 1:**
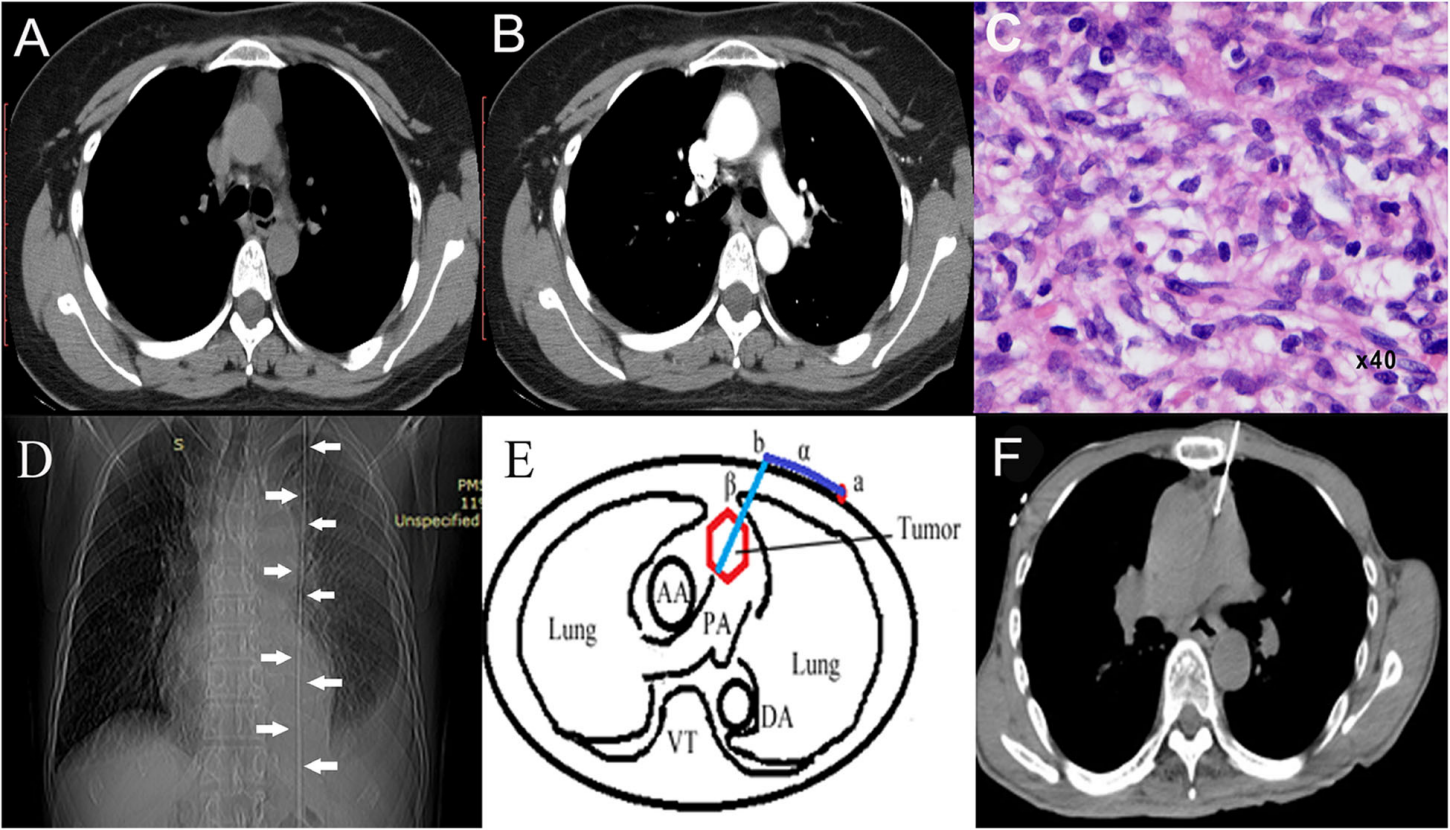
Puncture pathway design. **a, b** and **c** are preoperative CT images for mapping the mass and the related structure around the mass. **a** is a plain CT scan. **b** is the arterial phase of the scan, while **c** is in the venous phase of the scan. After mapping the mass, an imaging marker was placed on the surface of the body (white arrows in **d**, denoted by a in the transverse section). Illustration of the design process for the puncture approach (light blue line, denoted by β) and calculation of the insertion site (red point, denoted by b) is shown in **e**. The blue line between **a** and **b** is the distance from the imaging marker (**a**) to the insertion site (**b**). β is the depth of the puncture into the tumor (red hexagon). To ensure complete ablation, a sufficient puncture depth into the mass was essential. After careful design and step-by-step insertion, the radiofrequency probe (white line) was finally placed at the planned position in the tumor (**f**)

The RFA procedure was performed under CT guidance. The RFA path was designed through the preoperative plain CT images according to the tumor position. The anterior approaches were generally selected in a lateral position or supine position. The tract passed through the chest wall and/or partial peripheral lung tissue to reach the thymus mass without passing through any vessels and nerves (Fig. 1). This procedure requires accuracy to minimize the possibility of damage to the major vessels, principal bronchus, and nerves along the puncture pathway.

### RFA procedure

The entire procedure was performed on a 16-slice PHILIPS Brilliance TM Big Bore CT scanner (Koninklijke Philips N.V., Amsterdam, Netherlands). Before the operation, patients were placed on the CT bed in a lateral or dorsal position. When the position was confirmed, the patient remained still during the procedure for an accurate puncture. A slim galvanized wire was applied on the surface of the skin along the body as an imaging marker. The wire was used to locate the puncture (white arrows, D in Fig. 1) since the wire is visible on CT images. A plain CT scan was performed to locate the mass and design the puncture pathways with the help of the imaging marker. To clearly visualize the vessels that were involved within the procedure area, it was essential to intravenously inject a contrast agent. Sequential scanning, for both plain and contrast-enhanced CT scans, was performed using a 5 mm slice thickness and slice gap. With a similar method, the operator designed the puncture pathway of the monopolar RFA probe (17-15s30F, STAR med Co., Ltd., GYEONGGL-DO, Korea) and marked the puncture site on the skin (Fig. 1).

**Fig. 2:**
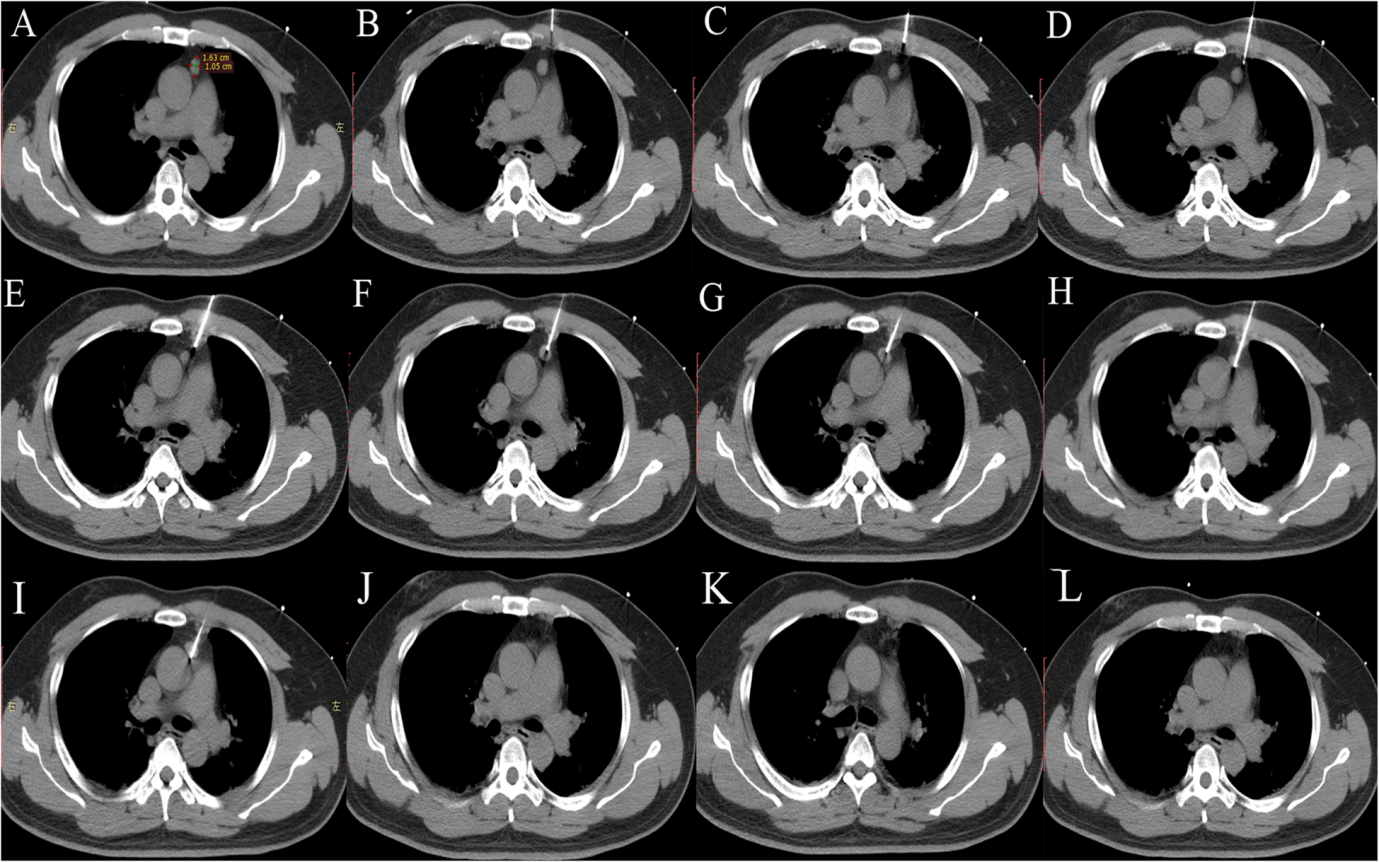
Illustration of the step-by-step puncture procedure and appearance immediately after ablation. The size of the thymoma was included to calculate the ablation time before puncture (**a**). After designing the puncture approach, the step-by-step puncture was performed from the surface to the tumor, as shown (**b-i**). Immediately after ablation, an additional CT scan was performed to identify residual lesion tissue (**j, k** and **l**). The lesion was completely ablated

When the procedure started, intravenous anesthesia (remifentanil, 0.05–0.1 μg/kg/min) was administered, and the vital signs of the patient were closely monitored by the anesthetists. After disinfection, a subcutaneous injection of lidocaine was administered at the puncture site. A 17 G probe was inserted step-by-step from the puncture site following the planned puncture pathway (Fig. 2). Repetitive plain scanning followed each step of the insertion during the step-by-step procedure to adjust the probe to the planned pathway. When the probe was placed in the perfect position, the ablation was started. The length of the ablation time was mainly determined by the size of the mass.

**Fig. 3:**
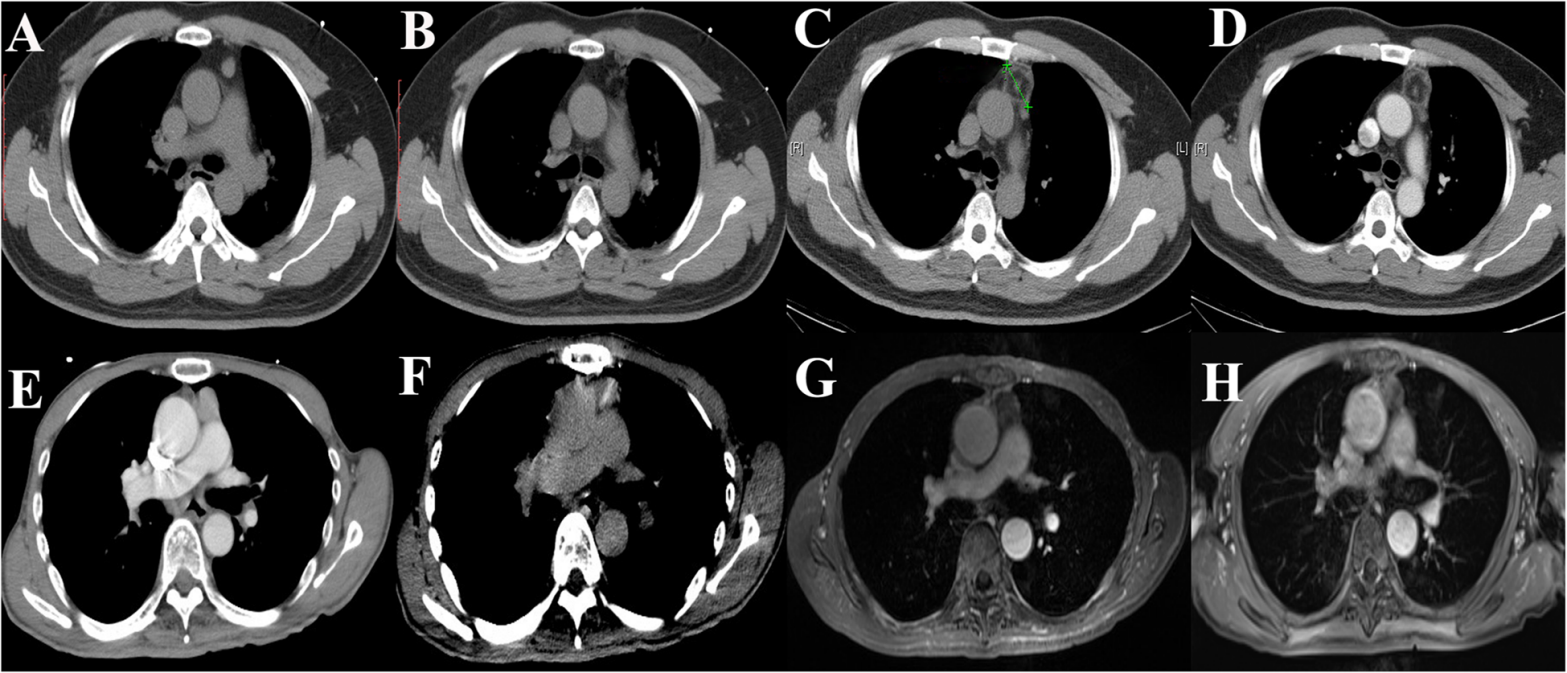
Follow-ups after ablation. The images in the first row belong to one patient, and the images in the second row are from another patient. **a** and **e** are the preoperative images. **b** and **f** are images immediately after ablation. **c** and **g** are the follow-up images one month later. There was no residual lesion tissue or recurrence. **d** is the follow-up image three months later. **h** is the follow-up image one year later. There was no residual lesion tissue or recurrence. Routine follow-up was essential for the early detection of recurrences

When the ablation was completed, contrast-enhanced CT scanning was performed to map the residual lesion(s). If the ablated margin surrounding the tumor was < 5 mm, the ablation time was prolonged. After complete ablation was confirmed, coagulation was performed along the needle tract before the probe was removed to prevent metastasis along the tract. After the procedure, an additional CT scan was performed to ensure that there were no signs of bleeding or pneumothorax (Fig. 3).

### Follow-up

Postoperative CT or MR scans, clinical examinations and blood work for follow-up were scheduled at 3–4 weeks, 3 months, 6 months, and 12 months postoperative and at intervals of once a year thereafter. The follow-up times were calculated from the day of intervention to the day of the last radiological examination or to the day of data harvesting. If there was any residual mass or local recurrence shown on the contrast-enhanced CT or MR images during the follow-up appointments, additional RFA was recommended. Local recurrence was defined as new lesions that emerged adjacent to the ablated area.

## Results

### Clinical characteristics

The data of patients were retrospectively analyzed and are presented in Table 1. In the current study, the ratio of males to females was 0.85/1.0. Myasthenia gravis was present in approximately half of all patients (6/13, 46.2%). Based on the 2004 World Health Organization (WHO) histological criteria [16], type A thymoma was the most commonly observed subtype (46.1%).

### Treatment effectiveness

All ablations were performed by the radiologist under CT guidance with the 16-slice PHILIPS Brilliance TM Big Bore CT. The average operation time was 52 min (ranging from 41 to 67 min), while the average time for ablation was only 15 min (ranging from 10 to 20 min). Minimum blood loss was involved. The average hospital length of stay was 4 days (ranging from 3 to 6 days). All lesions were completely ablated (13/13, 100%) based on the CT scans that were acquired immediately after RFA (Fig. 2j, k, l). The median follow-up time was 80.5 months (ranging from 64.6 to 116.9 months). Regarding the follow-up appointment observations, only one out of the thirteen patients had recurrence at 35.5 months after the initial ablation (1/13, 7.7%). The patient with recurrence was immediately examined to determine the optimal treatment. The details are shown in Table 2.

**Table 2:** Effectiveness of RFA treatment in the patients with thymomas

Patient No.	Ablation time (min)	Operation time (min)	Hospitalization time (days)	Cost ($)	Follow- up time (months)	Recurrence	Recurrence time (months)
1	20	51	4	1808	64.6	N	–
2	15	44	5	1632	67.2	N	–
3	15	62	5	1935	116.9	N	–
4	20	45	3	1825	81.6	N	–
5	12	52	3	1739	77.5	N	–
6	18	41	4	1689	101	N	–
7	12	63	3	1843	71.3	N	–
8	15	45	4	1860	98.1	Y	35.5
9	14	60	5	1768	75.8	N	–
10	10	67	6	1926	80.3	N	–
11	15	52	3	1765	91.4	N	–
12	12	64	3	1846	84.4	N	–
13	10	62	5	1982	80.5	N	–

### Complications

There was one major complication recorded. One patient who had puncture-related bleeding that needed a blood transfusion and intravascular embolization of injured vessel. There were no procedure-related deaths after RFA. The most frequent postoperative complications reported were fever, incisional pain, fatigue, nausea, pleural effusion, pneumothorax and hemoptysis. Grade I or II fever after ablation emerged in all patients (13/13, 100%). Three patients developed mild incisional pain (23.1%), and ten patients (76.9%) developed moderate incisional pain. The pain in all patients was alleviated after pain management; four patients (30.8%) developed grade I nausea after ablation, and one patient (7.7%) developed grade II nausea. Minor pleural effusion was reported in two patients (15.4%). Three patients (23.1%) developed mild pneumothorax. These complications could be easily controlled with symptomatic treatments and usually disappeared within 1 to 4 days.

## Discussion

For patients with small stage I thymoma, open surgical resection is considered to have a high risk of potential surgical injuries and other complications. Therefore, some patients with small tumors may think that open surgical resection would not be worthwhile. For those patients, treatment methods that causes minimal trauma are desired. However, there are no guidelines regarding which patients might be the best candidates for CT-guided radiofrequency ablation. The patients should be carefully selected based on their willingness and readiness to undergo RFA. In addition, some patients refused surgical resection for other reasons, such as economical, physical, and psychological factors.

In the current study, radiofrequency ablation was shown to be a curative treatment that causes minimal trauma in stage I thymoma patients. The current study reported 13 patients with small masses diagnosed as stage I thymoma. The ratio of males to females was 0.85/1.0, which is consistent with the ratio in previous reports [17–19]. Six of the patients diagnosed with thymoma had coexisting myasthenia gravis (6/13, 46.2%). Myasthenia gravis might help diagnose early-stage thymoma, especially for patients who have small masses that cannot be detected in routine examination. The smallest mass in the current study was 16.3 mm. The average size was 31.5 mm (ranging from 16.3 to 43.5 mm). Regarding the prognosis after ablation, thymomas of histological subtypes A, AB and B1 had low malignant potential.

Compared to the results of previous reports [3, 18, 20], there are several notable points:

First, the RFA procedure time is generally shorter than that for surgical resection. In the current study, the average operation time was only 52.0 min. Only 15.0 min (average time) was needed for ablation. In addition, a combination of intravenous sedation and local anesthesia was administered during the operation instead of general anesthesia. A short operation time and regional intravenous anesthesia could lead to fewer postoperative complications than the use of general anesthesia in surgical resection. The average length of hospital stay was shortened to approximately only 4 days, and there was a lower cost associated with RFA than with surgical resection.

Second, RFA was performed under CT guidance and does not require open chest surgery. Although video-assisted thoracoscopic surgery causes minor trauma and has a clear surgical view, complete resection under VATS is a major challenge for most surgeons. However, CT-guided radiofrequency ablation can be easily performed by surgeons and radiologists.

Third, the average follow-up time was 80.5 months, ranging from 64.6 months to 116.9 months. One patient (7.7%) had recurrence during follow-up at 35.5 months after the ablation, but the patient was cured after additional RFA. The 5-year overall survival rate was 100% (13/13), while the 5-year disease-free survival rate was 92.3% (12/13).

Fourth, there was one patient who accidentally suffered one major complication secondary to severe puncture-related bleeding during the operation and who needed blood transfusion and intravascular embolization of the punctured-injured vessel (1/13, 7.7%). The hemoptysis after operation was generally mild, and it could be resolved with symptom management. However, there are potential risks of puncture-related bleeding in this procedure, which greatly depends on the operator’s experience. This complication was mainly caused by a puncture injury to a small blood vessel in the planned pathway in the lung. Maintaining accuracy along the puncture pathway and a slow insertion are essential points to minimizing injury. The closer the puncture pathway is to the peripheral pulmonary lobe, the higher the risk of injury. If an accidental injury to a large vessel causes puncture-related bleeding that is life threatening, intravascular embolization might control the bleeding. Moderate puncture-related pneumothorax after ablation could be alleviated by thoracic closed drainage. The other common complications were mild and were mainly due to necrotic tissue present after ablation and anesthetics. Serious adverse events were not observed in the current study, and complications could be resolved with symptom management.

## Conclusions

In conclusion, CT-guided RFA applied in early-stage I thymoma as a curative treatment is feasible and has the advantages of minimal risk of injury, low cost, low rate of complications, and excellent complete ablation rate. Although the patient sample size was small, the current study indicates the feasibility of achieving complete ablation of thymoma by CT-guided RFA and its advantages over surgical resection. The conclusions of the current study need to be confirmed in a larger cohort, but suggest that RFA is a potential novel solution for patients with early-stage thymoma.

## Abbreviations

CT: Computed tomography
ECOG: East Coast Oncology Group
FNC: Fine-needle cytology
MG: Myasthenia gravis
MRI: Magnetic resonance imaging
RFA: Radiofrequency ablation
VATS: Video-assisted thoracoscopic surgery
WHO: World Health Organization
